# Laparoscopic splenectomy both for primary cytoreductive surgery for advanced ovarian cancer and for secondary surgery for isolated spleen recurrence: feasibility and technique

**DOI:** 10.1186/s12893-021-01368-z

**Published:** 2021-10-28

**Authors:** Antonio Macciò, Elisabetta Sanna, Fabrizio Lavra, Giacomo Chiappe, Marco Petrillo, Clelia Madeddu

**Affiliations:** 1 Department of Gynecologic Oncology, ARNAS G. Brotzu, Cagliari, Italy; 2 Department of Surgical Sciences, University of Cagliari, Cagliari, Italy; 3 Gynecologic and Obstetric Unit, Department of Medical, Surgical and Experimental Sciences, University of Sassari, Sassari, Italy; 4 Department of Medical Sciences and Public Health, University of Cagliari, Cagliari, Italy

**Keywords:** Laparoscopy, Splenectomy, Ovarian cancer, Platinum-sensitive disease, Quality of life, Minimally invasive surgery

## Abstract

**Background:**

This study investigated the feasibility and safety of laparoscopic splenectomy conducted in the contexts of both laparoscopic secondary surgery for isolated recurrence in the spleen and primary laparoscopic cytoreductive surgery for advanced ovarian cancer.

**Methods:**

We performed a perspective observational study including all consecutive patients with ovarian cancer who underwent laparoscopic splenectomy as part of primary cytoreductive procedures for advanced stage ovarian cancer or secondary surgery for isolated splenic recurrence between January 2016 and May 2020.

**Results:**

We enrolled 13 consecutive patients, candidate to laparoscopic splenectomy as part of primary cytoreductive procedures for advanced stage ovarian cancer (6 patients) or secondary surgery for isolated splenic recurrence of platinum-sensitive ovarian cancer (7 patients). Median operative time (509 min [range, 200–845]) for primary cytoreductive surgery varied according to surgical complexity depending on the extensiveness of the disease. Median operative time for secondary surgery for isolated splenic metastasis was 253 min (90–380). Only 1 patient with isolated splenic recurrence required conversion to an open approach. No intraoperative complication occurred, and no intraoperative blood transfusions were required. Median hospital stay was 3 days (range, 2–5) for isolated recurrence and 9 days (7–18) for primary cytoreductive surgery. Complete tumor resection was achieved in all patients. Median time from surgery to adjuvant chemotherapy was 16 days (7–24). All six patients who underwent laparoscopic splenectomy during primary cytoreductive surgery remain alive, four of whom exhibit no evidence of disease (median follow-up 25 months [4–36]). Among patients who underwent laparoscopic splenectomy during secondary surgery for isolated splenic relapse, all patients are alive and only one had a central diaphragmatic relapse 2 years after surgery (median follow-up 17 months ([5–48 months]).

**Conclusions:**

The laparoscopic approach to splenectomy is feasible and safe both in patients undergoing primary cytoreductive surgery for advanced stage disease and those with isolated recurrence of ovarian cancer, without compromising survival and allowing early initiation of postoperative systemic chemotherapy.

**Supplementary Information:**

The online version contains supplementary material available at 10.1186/s12893-021-01368-z.

## Background

Splenectomy through an abdominal open approach combined with upper abdominal surgery has become a crucial component of cytoreductive surgery for advanced ovarian cancer [1–4]. More recently, the possibility of performing this surgical procedure using a minimally invasive approach has been investigated [5, 6].

To date, laparoscopic splenectomy has been utilized mainly as a potential surgical option for the treatment of isolated recurrence of platinum-sensitive ovarian cancer in the spleen and has been shown to be a feasible intervention in the hands of a gynecologic oncology surgeon with expertise in minimally invasive surgery [7–15]. Conversely, laparoscopic splenectomy in cases of primary surgery for advanced tumor has not been well investigated. Indeed, laparoscopic splenectomy as primary surgery for advanced disease is a more complex surgery in which aggressive optimal cytoreductive surgery does not only include the isolated surgical time on the spleen, since the peritoneum, omentum, and diaphragm are often involved in the neoplastic process [16].

The purpose of this study was to demonstrate the feasibility and safety of laparoscopic splenectomy carried out in the contexts of both laparoscopic secondary surgery for isolated recurrence in the spleen and primary laparoscopic cytoreductive surgery for advanced ovarian cancer, where the fundamental goal is the optimal radical cytoreduction. The different technical issues involved are highlighted by reporting three emblematic cases and providing corresponding surgical videos.

## Patients and methods

Here we prospectively analyzed all consecutive patients with ovarian cancer who underwent laparoscopic splenectomy as part of primary cytoreductive procedures for advanced stage ovarian cancer or secondary surgery for isolated splenic recurrence from January 2016 to May 2020 at the Department of Gynecologic Oncology, Businco Hospital, ARNAS G. Brotzu. Among 172 patients who underwent laparoscopic surgery for ovarian cancer, 13 patients underwent laparoscopic splenectomies, seven for an isolated splenic recurrence; all procedures were performed in patients highly motivated for minimally invasive surgery/approach. Here we also report the detailed videos of three emblematic patients. One of the three patients underwent laparoscopic splenectomy during secondary surgery for platinum-sensitive recurrent disease with an isolated splenic lesion and the other two patients as a component of primary cytoreductive surgery for advanced stage disease.

The study was performed in accordance with our institutional ethics committee guidelines and the principles of the Helsinki Declaration. In accordance with the Italian Regulatory Agency for observational studies not involving drugs and considering that the surgical procedure used were not experimental, the study has been notified and approved by the Institutional Independent Ethics Committee of the “Azienda Ospedaliera Universitaria di Cagliari”, Cagliari, Italy. Written informed consent was obtained from each patient for surgery as well as for the publication of the case report and the accompanying videos/images. Patient demographic, surgical, postoperative, and follow-up data were obtained from patient charts. Before surgery in each case, we evaluated the extent of disease or pattern and precise localization of recurrence of disease by computed tomography (CT) and 2-deoxy-2F-^18^fluoro-d-glucose (FDG) positron emission tomography (PET)/CT.

Eligibility criteria were: good clinical performance status (Eastern Cooperative Oncologic Group performance status 0–2); stable medical condition and absence of comorbidities that contraindicate laparoscopy; patient motivation for a minimally invasive approach; initial diagnostic laparoscopic assessment that confirmed the operability with possibility of optimal laparoscopic cytoreduction with a goal of no residual disease (laparoscopic criteria are specified in the paragraph “surgical technique”). For patient candidate to secondary cytoreductive surgery for isolated recurrent disease, specific eligibility criteria were: platinum-free interval ≥ 12 months; isolated pattern of recurrence with absence of disease in other region of the peritoneal cavity as assessed by precise radiologic localization of recurrent disease using positron emission tomography computed tomography (PET-CT) before surgery.

Exclusion criteria were high anesthesiologic risk (ASA III or more), and any preoperative contraindication to laparoscopy, i.e., reduction in the respiratory capacity, inability to tolerate Trendelenburg position or pneumoperitoneum throughout the entire surgical procedure; severe adherential syndrome with obstacle to organ access, resulting from previous surgery, i.e., previous extensive peritonectomies, which can impede an optimal laparoscopic approach.

Data were collected on disease stage according to the International Federation of Gynecology and Obstetrics (FIGO) system at the time of primary cytoreduction, type of primary surgery, histological type and grade, operating time, estimated blood loss, length of hospital stay, and residual tumor at the end of surgery. The intraoperative and early postoperative complications were recorded according to the Clavien-Dindo classification [17].

The patients were discharged when they were medically stable and able to tolerate oral intake. All patients received vaccination for *Streptococcus pneumoniae*, *Haemophilus influenzae*, and *Neisseria meningitidis*. All patients underwent a PET-CT scan by 1 month after surgery. Then, standard adjuvant chemotherapy was started as soon as tolerated within 30 days of surgery.

### Surgical technique

All procedures were performed by the same senior surgeon (AM) who has extensive training and experience in both gynecologic oncology and minimally invasive surgery of the pelvis and upper abdomen.

Perioperative antibiotic therapy and postoperative thromboembolic prophylaxis were used routinely. In cases of advanced-stage cancer, thromboembolic prophylaxis with low molecular weight heparin was started at the time of diagnosis of advanced ovarian cancer if fibrinogen and C-reactive protein values were indicative of an IL-6-correlated pro-thrombotic status [18].

A nasogastric tube was inserted due to the risk of gastric distention and perforation. Correct patient positioning in aggressive laparoscopic surgery (of the upper abdomen) is a critical step and vary depending on the specific techniques chosen by the operator and the anatomic characteristics of the patient.

We used an anterograde approach, with the first operator positioned between the patient's legs and the patient in supine position with the left side up to afford a better view of the organ at the time of splenectomy. The choice of such approach as well as the sequence of the operative steps, that are described in detail in the videos, may render our technique different form that utilized for laparoscopic splenectomy for the most typical indications (as autoimmune disease, splenomegaly due to hematological disease, etc..) and depends from the fact that the procedure in case of laparoscopic surgery for ovarian cancer, also in case of isolated recurrence, may not be limited to splenectomy alone and the need of additional surgical times cannot be excluded “a priori”. One of the challenges when attempting to perform an optimal cytoreduction by laparoscopic surgery is the potentially limited visualization of the upper abdomen; this is one reason for use of the 30° scope coupled with the left side elevation or table placing in moderate to steep reverse Trendelenburg position to allow excellent visualization of the spleen.

**Fig. 1:**
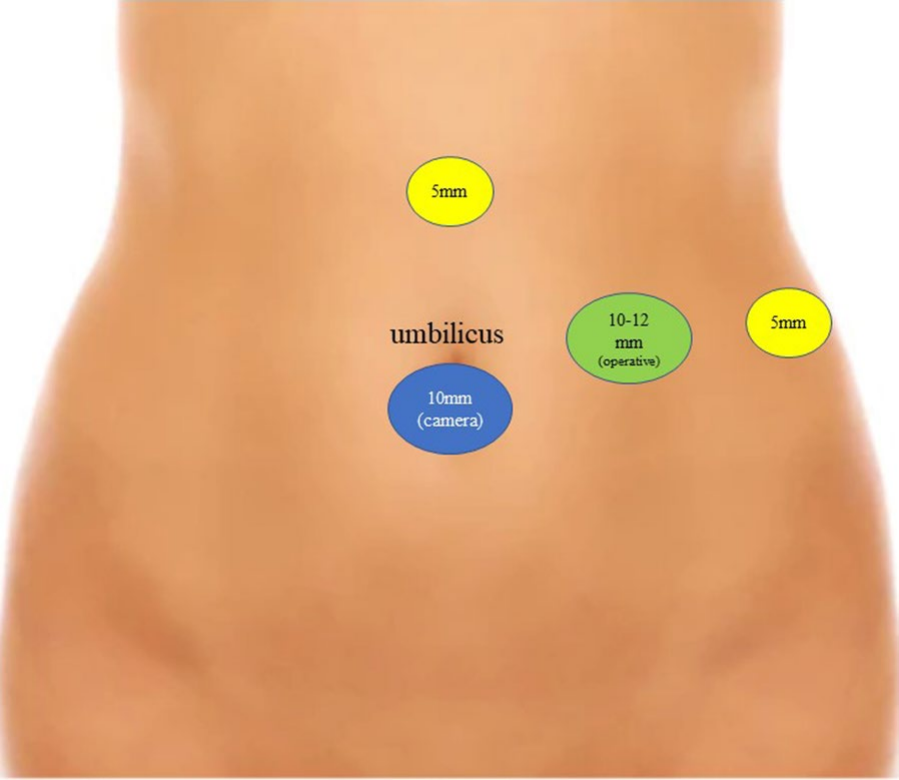
Iconographic description of port placement in ovarian cancer patients receiving laparoscopic splenectomy

The initial abdominal entry was performed using the open Hasson technique. All procedures were performed using a periumbilical laparoscopic approach with a 10 mm trocar for the camera; the camera trocar positioning could vary according to the anthropometric characteristic of the patient. An angled 30° 100 mm laparoscope was used. Additional two abdominal ancillary 5 mm trocars and one 10-12 mm trocar were placed in the lower and upper abdomen after careful intraoperative consideration (Fig. 1).

The secondary cytoreductive surgery procedure included initial careful diagnostic laparoscopic inspection of the abdomen and pelvis to localize other sites of disease and to confirm the absence of carcinomatosis and/or other neoplastic lesions. Also, in all women with advanced-stage disease, the possibility of optimal cytoreduction with the goal of no residual disease was evaluated by initial diagnostic laparoscopy and staging of the peritoneal index according to the rules proposed by Fagotti et al. in 2006 [19] and updated by Petrillo et al. in 2015 [20].

Various laparoscopic instruments and techniques may be used in this type of surgery, including sharp scissors or bipolar graspers and a Ligasure system (Medtronic-Covidien, Dublin, Ireland), endoscopic Ligaclips (Ethicon, Somerville, NJ, USA), and Endo GIA vascular staples (Johnson & Johnson Medical Devices, Irvine, CA, USA). Splenic mobilization is a central issue when performing this surgery. Laparoscopic splenectomy involves dissection of the four splenic ligaments; the order of dissection varies according to the preferences of the surgeon and, in the event of primary surgery for advanced stages, the specific spread of the disease. In fact, in the patients who underwent primary surgery for advanced ovarian cancer, the intervention was made more complex by the presence of malignant tissue in the omentum and diaphragm. The videos describe the technical modalities used, including those used to control the hilar vessels. Once the spleen was excised, all specimens were removed in an endoscopic bag through a slight enlargement of the umbilical incision. In the patients who underwent primary laparoscopic surgery for advanced cancer, the bag containing the spleen could also be removed through the vagina.

Antibiotic treatment was routinely continued for 3 or 4 days after surgery. The abdominal drain was usually removed after resumption of oral intake but only if a pancreatic fistula was not suspected.

Four additional videos accompanying this article illustrate the step-by-step surgical technique for laparoscopic splenectomy in the setting of secondary cytoreductive surgery with an isolated splenic metastasis for recurrent platinum-sensitive ovarian cancer (see Additional file 1: Video 1 and Additional file 2: Video 2) and in the setting of laparoscopic primary cytoreductive surgery for advanced ovarian cancer (see Additional file 3: Video 3 and Additional file 4: Video 4).

## Results

The patients’ clinical characteristics are reported in Table 1. In patients with isolated recurrence in the spleen, the median progression-free interval before surgery was 23 (range 12–34) months. One patient with an isolated splenic recurrence needed conversion to an open approach; the reason was a bleeding that cannot be completely controlled laparoscopically. The median estimated blood loss was 200 mL in primary cytoreductive surgery and 50 mL in secondary surgery. In case of laparoscopic primary cytoreductive surgery splenectomy was included as part of optimal cytoreduction including hysterectomy (6 cases), omentectomy (all cases), partial or total peritonectomy (all cases), diaphragmatic peritonectomy (4 cases), bowel resection with anastomosis (4 cases), and paraaortic lymphadenectomy (6 cases); therefore, the median operating time varied according to the extent of neoplastic disease and the organs involved, which conditioned the complexity of surgery; the time ranged from 200 to 845 min with a median operative time of 509 min. The median operative time for secondary surgery for isolated splenic metastasis was 253 min (range 90–380). No intraoperative complications occurred, and no intraoperative blood transfusions were required. The median hospital stay was 3 (range, 2–5) days for isolated recurrence and 9 (5–18) days for primary cytoreductive surgery. All patients who underwent primary surgery for advanced ovarian cancer received intraperitoneal chemotherapy with cisplatin 80 mg/m^2^.

**Table 1:** Patients’ clinicopathological characteristics and treatment details

Characteristics	Primary cytoreduction	Secondary cytoreduction
All cases, n	6	7
Age, years median (range)	55.4 (40–79)	58.8 (49–73)
FIGO stage		
III C	4	5
IV	2	2
Tumor histotype		
Serous	6	7
Endometriod	0	0
Tumor grade		
G1	0	0
G2–3	6	7
No residual tumor at primary debulking surgery	6	5
PFI-1, months, median (range)	25 (4–36)	23 (12–34)
Estimated blood loss, mL, median (range)	200 (80–550)	50 (30–200)
Operative time, min, median (range)	509 (200–845)	253 (90–380)
Hospital stay, days, median (range)	9 (5–18)	3 (2–5)
PFI-2, months, median (range)	NA	Median not reached

*FIGO* International Federation of Gynecology and Obstetrics, *PFI-1* primary platinum-free interval after primary cytoreductive surgery, *PFI-2* primary platinum-free interval after secondary cytoreductive surgery

Complete tumor resection was achieved, and splenic metastasis was confirmed by pathologic examination in all patients. Among patients who received primary cytoreductive surgery, histologic examination revealed hilar involvement in 4 patients and parenchymal metastases in 1 patient; among those with isolated splenic recurrence 2 patients had hilar involvement and 5 patients had parenchymal metastases. The median time from surgery to starting adjuvant chemotherapy was 16 days (range 7–24). One patient developed a febrile abdominal collection 48 h after surgery that resolved with laparoscopic washing of the splenic bed; the patient was discharged immediately after resolution. No further postoperative complications were observed. A postoperative FDG PET-CT scan was performed in all women by 1 month after surgery, and there was no persistence of pathologic uptake in any case.

Among 6 patients who underwent laparoscopic splenectomy during primary cytoreductive surgery, all patients remain alive, 4 of whom have no evidence of disease after a median follow-up time of 25 months (range 4–36). Among patients who underwent laparoscopic splenectomy during secondary surgery for isolated splenic relapse, all patients are alive, and only one had a central diaphragmatic relapse 2 years after surgery, with a median follow-up time of 17 months (range 5–48 months). Median survival time has not been reached yet.

## Discussion

This case series confirms the feasibility and safety of laparoscopic splenectomy both in secondary cytoreductive surgery for localized recurrent ovarian cancer in the spleen as well as supports its use in the context of aggressive maximal primary cytoreductive surgery for advanced ovarian cancer.

Optimal cytoreductive surgery for ovarian cancer may need to be quite extensive to achieve a minimal residual tumor load, which is considered the most important favorable prognostic factor [21] and may include the use of extensive upper abdominal surgery. Indeed, in accordance with the National Comprehensive Cancer Network guidelines, to achieve optimal surgical cytoreduction for ovarian cancer, removal of the relevant abdominal organs, including splenectomy, in addition to radical pelvic dissection, bowel resection, and/or appendectomy, resection of the diaphragm or stripping of another peritoneal surface, partial hepatectomy, partial gastrectomy, partial cystectomy and/or ureteroneocystostomy, cholecystectomy, and/or distal pancreatectomy should be considered (https://www.nccn.org/professionals/physician_gls/pdf/ovarian_blocks.pdf, accessed on October 20, 2020). Therefore, splenectomy has been widely recognized as a surgical procedure to be included to achieve optimal cytoreductive surgery in cases of advanced ovarian cancer with bulky upper abdominal disease [1, 2, 6, 22–27]. However, to our knowledge, the feasibility and safety of laparoscopic splenectomy within the primary radical cytoreductive surgery for advanced ovarian cancer has been very poorly investigated. Only 4 laparoscopic approaches have been reported in the largest series published to date on 260 women who underwent splenectomy for the management of splenic metastasis during cytoreductive surgery for advanced or recurrent ovarian cancer [28]. Therefore, our manuscript is intended to be a feasibility study within the very few studies available to date on laparoscopic surgery for the primary optimal cytoreduction of advanced ovarian cancer [10, 29–31]. These studies also included patients with upper abdomen-related disease and reported also some splenectomies performed as in our cases with the goal to obtain optimal cytoreduction: they showed that optimal cytoreduction is possible in most cases (reaching 95.3%, with 85.9% of cases of no residue). In this setting a recent review published by a leading international group assessed the role to laparoscopy in primary surgery for advanced ovarian cancer [32]. The authors concluded that the studies on laparoscopic surgery of advanced ovarian cancer, although limited, report an overlap of oncological outcomes compared to traditional surgery and highlighted how the selection of patients is a crucial point to reach a successful minimally invasive surgical treatment, taking into account surgical complexity and surgical adequacy.

In contrast, most evidence supporting laparoscopic splenectomy derives from case reports and case series in patients with isolated splenic recurrence in the context of secondary cytoreductive surgery [7–15]*.* These studies demonstrated the feasibility and safety as well as the usefulness of laparoscopic removal of an isolated splenic recurrence during secondary cytoreductive surgery for improving the prognosis, particularly in patients with platinum-sensitive ovarian cancer. There is a direct correlation between the platinum-free interval and post-relapse survival, and secondary cytoreductive surgery for localized relapse may offer further clinical benefit. Early diagnosis and exact anatomic localization of the recurrence are important when tailoring treatment to the individual patient. To date, the largest series of laparoscopic splenectomies performed in patients with platinum-sensitive recurrent ovarian cancer with an isolated splenic relapse was published by Gallotta et al. [7]. In that study, the patients (n = 8) had a short median hospital stay with limited intraoperative estimated blood loss and only one complication. Our results are superimposable on those reported by Gallotta et al. [7] and confirm that laparoscopic splenectomy performed by a team of oncologic gynecologists is a feasible and safe intervention in the context of second-instance surgery for an isolated splenic relapse.

Of relevance, our study also shows that laparoscopic splenectomy is a feasible and safe intervention in the more complex context of primary cytoreductive surgery for advanced disease, where obviously the fundamental goal is the optimal radical cytoreduction. In particular, in cases of advanced ovarian cancer, the involvement of the spleen is often associated with the involvement of omentum and diaphragm. Thus, splenectomy must be associated with the other surgical times needed to obtain optimal cytoreduction, such as the removal of the diaphragmatic peritoneum and of the metastatic omentum. Therefore, the complexity of laparoscopic splenectomy is further complicated because it must be preceded by the complex timing of the omentectomy, which can be extremely difficult because cancer can seriously affect the omentum. In addition, the left diaphragm can be involved extensively in the cancer process and, therefore, diaphragmatic peritonectomy represents a fundamental surgical time for the preparation of adequate surgical spaces and for identification of the correct anatomical references. In this regard, the videos included with the present article briefly show the surgical steps of omentectomy and removal of the left diaphragmatic peritoneum; these videos highlight the importance of these surgical steps for safe and effective splenectomy. It should be also emphasized that splenectomy is reasonable if carried out in the context of an optimal cytoreductive surgery, thus other surgical procedures useful for obtaining optimal cytoreduction are all crucial steps to provide meaning to the splenectomy. Therefore, laparoscopic management of splenectomy and upper abdominal disease alongside intestinal, ureteral, and retroperitoneal involvement in the primary surgery for advanced ovarian cancer is enabled by surgeons proficient in advanced laparoscopic techniques [6] and with an adequate learning curve [33].

In this context, it should be noted that, although apparently technically difficult in this highly complex anatomic region, a laparoscopic approach with a magnified view is likely to be associated with a more precise dissection. Moreover, the use of the most modern multifunction technical devices may help to reduce the degree of difficulty and improve the quality of surgery, possibly leading to more widespread use of minimally invasive surgery in this clinical setting. From a technical point of view, despite the innovations in technique and equipment, some concerns may still arise in such surgery, especially regarding dissection of the splenic hilum. Here, we performed splenectomies with an anterior approach to maintain complete vascular control of the splenic vessels and thereby decrease the risk of pancreatic injury. As shown by the videos included in this article, the techniques used to isolate and clamp the vessels may vary, mainly according to the anatomic characteristics of the individual patient, and in the case of advanced cancer based on the extent of neoplastic disease and involvement of vascular structures in the neoplasm. The technique/approach may also vary according to the instruments used, such as, the Ultracision Harmonic scalpel and Ligasure systems; the Ligasure systems have shown good feasibility and effectiveness in our experience. Thus, particularly in the advanced stages of disease, it is not possible to standardize the surgical approach, which remains a function of the extent of disease.

Beside the surgical-related outcomes and the contribution on specific technical issues, our study is relevant, especially since it supports the applicability of laparoscopic splenectomy in the context of a minimally invasive approach for a very advanced disease, especially in the context of primary cytoreductive surgery. In fact, in such setting of patients, it is of extreme relevance that a laparoscopic approach may positively affect the quality of life and the psychological well-being of patients, resulting in perception of a less disfiguring surgery and a more positive cosmetic impact, despite the extent/stage of disease [34]. In this regard, it is important to recognize the patient motivation for a minimally invasive approach, which can preserve body image integrity, which in turn is fundamental for maintaining the psychological integrity of these patients, particularly in the context of a very advanced stage of disease. This concept is a main objective in establishing our surgical approach in clinical practice, to the extent that we created the aphorism “The respect of the body is the respect of the soul”.

Another relevant issue favoring the choice of minimally invasive surgery is that such an approach, both in the context of primary and secondary cytoreductive surgery, allows a more rapid recovery and thus the initiation of chemotherapy sooner after surgery.

Our work remains a study of feasibility that could contribute to implement the development of next trials warranted to attribute a role to laparoscopy in advanced ovarian cancer, especially in the setting of primary optimal cytoreductive surgery.

## Conclusions

In conclusion, our findings show that the laparoscopic approach to splenectomy is feasible and safe in patients undergoing primary cytoreductive surgery for advanced stage disease as well as in those with isolated recurrence of ovarian cancer. Notably, the laparoscopic approach may have a positive physical, psychological, and spiritual impact, thus improving patients’ quality of life and global well-being, without compromising their survival while allowing early initiation of postoperative systemic chemotherapy.

## Supplementary Information

**Additional file 1: Video 1.** The video shows surgical steps of laparoscopic splenectomy for secondary cytoreduction in a woman with high-grade serous ovarian cancer with isolated splenic recurrence. WeTransfer link: https://we.tl/t-cQbFvSdsuH.

**Additional file 2: Video 2.** The video illustrates the main surgical steps of laparoscopic splenectomy in a patient with isolated splenic recurrence from platinum-sensitive ovarian cancer. WeTransfer link: https://we.tl/t-ey6VWUOU3U.

**Additional file 3: Video 3.** Laparoscopic splenectomy as a component of primary cytoreductive surgery for advanced ovarian cancer (stage IIIC). The video includes the step of diaphragmatic peritonectomy. WeTransfer link: https://we.tl/t-VTIVdMnuD4.

**Additional file 4: Video 4.** Laparoscopic splenectomy during primary cytoreductive surgery in advanced ovarian cancer (stage IIIC). The video includes the step of omentectomy. WeTransfer link: https://we.tl/t-Y3Jpu5TrR2.

## Data Availability

The datasets used and analyzed during the current study are available from the corresponding author on reasonable request.

## Abbreviations

CT: Computed tomography
FDG: 2-Deoxy-2F-^18^fluoro-d-glucose
PET: Positron emission tomography
FIGO: International Federation of Gynecology and Obstetrics
